# Meeting of the Mississippi Valley Dental Society

**Published:** 1874-04

**Authors:** 


### MEETING OF THE MISSISSIPPI VALLEY DENTAL SOCIETY.

The thirtieth annual meeting of this Society was held in the city of St. Louis, beginning on the fourth of March and continuing three days. Joint meetings were held during the time by this and the Missouri State Dental Society; the meetings were thereby rendered very interesting. There was a large number, perhaps about one hundred, in attendance.

The discussions upon the subjects presented were very good and instructive. We hope to give a synopsis of them in the next No. of the REGISTER. There was also quite a number of good papers read, which will appear in due time in the REGISTER and *Missouri Dental Journal.*

It seemed somewhat strange for the Miss. V. Society to meet away from its accustomed place. Quite a number of its old members were present, however; but many who have almost always been present were absent, and were much missed.

Though it is very pleasant, and perhaps profitable in some respects, to hold joint meetings of two or more of our regular professional societies, there are some respects in which it is objectionable. Far more time is consumed by mere business matters than when but one society is meeting, and to such an extent was this true here that the interest in the subjects for discussion was somewhat abridged. Then, again, in a joint meeting there is a diminished, or rather a sort of divided, responsibility on the part of the members. We would suggest that joint meetings be held, only when there is something special calling for them.

We found the profession in St. Louis fully sustaining their reputation for hospitality, and brotherly sociability and good feeling. Nobody can make them a visit without being made better by it; and, in addition to all this, St. Louis is a *magnificent city.*

